# Understanding social gym intimidation and anxiety using the power-threat-meaning framework

**DOI:** 10.3389/fspor.2026.1712367

**Published:** 2026-04-29

**Authors:** Francis Quinn

**Affiliations:** School of Law and Social Sciences, Robert Gordon University, Aberdeen, United Kingdom

**Keywords:** anxiety, gym, intimidation, power, power-threat-meaning framework

## Abstract

While many gymgoers do not experience psychological barriers to gym use, some feel anxious and intimidated there in the presence of other users. Qualitative research has suggested gyms can be places of unequal power leading to threats to one's adequacy of self, and privilege certain users based on demographics, masculinity, size, fitness, performance, competence, etc. Social comparison and trait social anxiety have been suggested as contributing to gym anxiety. However, a theoretical approach is needed to provide a coherent explanation of gym anxiety which can inform interventions; there is no current theory in use in the research literature. I argue that gym anxiety is not the same as social physique anxiety (a limited concern about physique evaluation) nor social anxiety (often conceptualized as a mental health “disorder” rather than a response to the norm-laden setting of the gym). I propose that the most suitable theoretical framework to explain gym anxiety is Johnstone and Boyle's Power-Threat-Meaning Framework (PTMF), designed to explain psychological problems or distress. Gym anxiety or intimidation does not represent a disorder or pathology but an understandable response among some users to the power differentials of the gym environment. The PTMF explains how the negative operation of social power, in combination with societal and personal meaning-making, can lead to a perception of subtle threats to one's psychological needs, such as exclusion, invalidation, or rejection; the resulting “symptoms” such as anxiety, shame, hypervigilance, and avoidance are conceptualized as coping strategies called threat responses. By addressing one or more of power imbalances, perception of threat, meaning-making and threat responses, interventions have the potential to make gym anxiety more manageable by addressing some of its root causes, either in individual interventions or at wider group level, including by changes that can be made to specific gym settings.

## Introduction

Physical exercise has many benefits for physical health and mental health. Regular exercise reduces risk of acute and chronic diseases, increases lifespan and years lived in good health, improves cognitive function and mood, and reduces anxiety and stress (1). But beyond health benefits, in some societies (especially but not exclusively in the West) it has become socially desirable to possess a body with certain characteristics, with supposed privileges (e.g., status, mate choice) for people whose bodies appear youthful, healthy, show high performance, low body fat, and (especially for men) high muscle mass (2). This has been seen by some social scientists as part of a problematic discourse found in Western societies of right and wrong ways to live, called “healthism”, in which health is the personal responsibility of each individual, to be improved via self-discipline, effort and investment of personal resources (time, money, etc.), with good health (and thus fitness) indicating one is a good citizen and the unhealthy or unfit blamed as lazy, a slob, couch potato etc. [e.g., (3, 4)].

In some countries, public exercise facilities such as gyms are widespread and a common choice for exercise. Operated on a non-profit or commercial basis, gyms offer advantages for exercisers such as more space and facilities than at home, alternative and tempting sedentary activities being unavailable, access to knowledgeable staff, and protection from inclement weather.

However, gyms can be challenging spaces for some users, or maybe even many users some of the time, due to feeling anxiety and/or intimidation about exercising there, including anticipating negative judgements from others (5, 6). This gym anxiety appears common; indeed, an internet search for terms such as “gym anxiety” and “gymtimidation” (the latter a trademark of U.S. company Planet Fitness) produces thousands of results and was even the focus of a Google billboard ad in the early 2020s about common questions inputted by users of the search engine. Gym anxiety acts as a barrier to gym attendance; for example, a 2025 market research survey among non-gym members representative of the UK population on age, sex, ethnicity and social class found that 47% endorsed “I feel uncomfortable at the thought of joining a gym or leisure facility”, 32% endorsed “I feel I would be negatively judged if I join”, and 17% endorsed “I would feel unsafe… due to the threat of sexual harassment or intimidation” (7). In a systematic review of adherence to exercise referral schemes among middle-aged and older adults, a major barrier participants reported was feeling intimidated and uncomfortable in gyms around other exercisers who were young, slim and fit (6), while among diverse inactive girls aged 11–14, a key barrier to exercise was feeling self-conscious about their appearance (8).

The present article provides a new insight into why some gym users feel anxious around other users, using the idea of social power and how people cope with the threats that power poses. I focus on where the feared situation is interpersonal in nature [i.e., not intimidation or anxiety that could occur if the exerciser was alone, such as concern that exercising might cause an injury or heart attack, or that one does not feel confident they can do what the activity requires to count as exercise, e.g., (9)]. In this article I use the terms “anxiety” and “intimidation” interchangeably; even if these two concepts are different in some way, I argue that they stem from the same causes.

### Gym intimidation is distinct from other concepts in the literature

There are concepts in psychology that overlap yet are not identical (35), with gym anxiety/intimidation, self-presentation, social physique anxiety and social anxiety being examples. In this article I treat gym anxiety and intimidation as separate to self-presentational concerns, social physique anxiety or social anxiety. I argue below that gym anxiety appears to be an understandable response to a difficult situation, rather than a manifestation of poor mental health (as with social anxiety) or primarily a self-presentational concern, and is specific to the gym setting (unlike social physique anxiety), and provide a non-pathologizing explanation for gym anxiety.

For over a century, social scientists have examined the human desire to present oneself in an appealing way in social situations, and to control how others perceive them. This *self-presentation* or impression management has been studied in the sport and exercise psychology literature (11), including to what degree a person is concerned about presenting a good or bad impression to others, and their confidence in making a desirable impression. Self-presentation is different from gym anxiety as it is a behavioral process that one engages in to control the impression made on others, unlike anxiety/intimidation which is a felt state.

Related to self-presentation, since the 1980s researchers have investigated *social physique anxiety*, where a person is apprehensive about their physique being evaluated by others negatively (12). This experience is not limited to gyms or even exercise settings but has largely been studied in the exercise psychology literature. Unsurprisingly, high levels of social physique anxiety have been associated with reduced physical activity in a range of age groups (13). However, sometimes no clear relationship has been found with physical activity; social physique anxiety can be a motive to exercise more, a deterrent from exercise, or both at the same time; therefore, the effect of social physique anxiety on individuals’ physical activity may cancel each other out in quantitative studies aggregated across participants because social physique anxiety makes some people exercise more, makes others avoid exercise, and others are conflicted. Social physique anxiety is increased by environments that draw attention to users’ physiques (e.g., tight-fitting clothes, mirrors, comments from instructors or trainers about exercising for physique rather than health) and is greater when exercising around same-age peers than around one's parents (14). However gym intimidation is experienced in relation to place and multiple potential threats as shown in research discussed below, rather than to a specific outcome as in social physique anxiety in which negative evaluation of the body is feared irrespective of location of exercise (e.g., whether on a day at the beach or exercising in the gym), and therefore merits a distinct conceptualization.

Another concept that might seem relevant is social anxiety, which is associated with situations in which one fears being evaluated negatively by others. Indeed, Levinson et al. (15) conceptualized social exercise anxiety as a trait, measured psychometrically and correlated with trait social anxiety. However, social anxiety is a concept that pathologizes the experience and has often been conceptualized as a psychiatric “disorder”, with theories explaining social anxiety often locating problems in the individual's cognitions, attentional bias and behaviors that reinforce a vicious cycle, such as Clark and Wells’ cognitive-behavioral model (16). This is not so applicable here because gym anxiety is specific to the gym environment, rather than generalized to other settings where the person is evaluated in some way (e.g., general social settings) as in psychiatric diagnoses such as “social anxiety disorder”. As literature below suggests, gym environments are not simply another social situation and are often loaded with norms, meanings and cultural expectations of how an exerciser should act, look and perform, rather than necessarily indicating poor mental health on the part of the user. Nor is gym anxiety generally associated with individual psychopathology as in common conceptualizations of social anxiety [e.g., (16)]. While people with social anxiety may be more likely to experience gym anxiety, this label does not seem appropriate for general use when problematic anxiety or intimidation is limited to gym settings.

### Gym intimidation as barrier to population physical activity and operator profitability

Gym intimidation can reduce how much exercise users do: exercisers who felt more intimidated reported reduced duration, exertion, frequency, and adherence, and gym users terminated exercise on an elliptical trainer sooner if others around them showed greater exertion (17). Additionally, affective expectancies about exercise (e.g., will feel good or bad) are triggered automatically; these play a key role in determining whether exercise is repeated, according to Brand and Ekkekakis's (11) affective-reflective theory (ART). According to ART, affective expectancies are based mainly on prior experiences of exercise, so exercise in a setting perceived as intimidating and threatening will quickly lead to negative affective expectancies, which lead to avoidance impulses, and lack of desire to repeat that exercise. The person is then unlikely to repeat that exercise unless self-control is very strong. This matters because the most effective exercise for health, fitness, or any other desired outcome, is whatever exercise that person is most likely to engage in consistently. While some intimidated gym users could exercise at home or in a different public setting, limitations of space, equipment, and staff there restrict the exercise goals possible, missing out on a gym's advantages.

Gym intimidation matters also to the growth and profitability of the fitness industry, as a barrier to customers signing up and continuing with memberships, affecting sales and retention. Many gym operators are aware of this, and some chains use slogans such as “Everybody Welcome” (PureGym, UK) or “No Lunks” (Planet Fitness, USA). As anxiety and intimidation are unpleasant, it is important that gym operators address intimidation because affect is central to customer loyalty to fitness facilities, especially for novice users; for example, in Japan, pleasure of using the facility predicted attitudinal loyalty to the gym operator as well as visit frequency (12).

### What gym situations are intimidating?

Intimidation around other exercisers is more likely to occur when the exerciser perceives they do not match the characteristics of the group(s) which seem to be more numerous, or the most dominant, in the gym space, and that members of these groups pose a perceived psychological or physical threat. In this context, often (but not always) these “dominant” gym users seem healthy, fit, slim, confident and competent at exercising, young, male, and if male, with traditionally masculine mannerisms. In addition, differences in body fat levels and performance, leading to anticipated ostracism, are common reported sources of intimidation.

Much of this research uses qualitative methods and has been conducted with minoritized participants in that society. For example, overweight Black women in disadvantaged areas of South Carolina, aged 25–50, experienced gym intimidation which they perceived was related to differences in their weight and body performance, compared to other exercisers and they had experienced cruel comments from others in the gym (13). In Chicago, low-income Black women feared being teased as unfit if exercising in public, which they believed would harm their self-esteem (18). And among young women diagnosed with anxiety disorders, social evaluation by others about quality of their exercise technique and sweaty appearance were attributed as contributing to anxiety and worry about exercise, leading to avoidance of exercise (19).

Furthermore, adults with physical disabilities anticipated fitness centers as an exclusive space with negative attitudes toward them from other gym users (20). In patients diagnosed with COPD, who used a public gym for pulmonary rehabilitation, intimidation and embarrassment occurred when they struggled to use gym equipment and perceived themselves as standing out as a result (21). Finally, an ethnically diverse and physically active group of able-bodied American undergraduate women in a campus gym experienced greatest “self-consciousness” and “discomfort” at the gym when the facility was crowded, other exercisers were perceived to be very fit, men watched them overtly, and they perceived they lacked competence at exercising (22).

Surveys with large samples have also shed light on the kinds of situations that are most intimidating: as with the qualitative studies above, this tends to be when not meeting perceived standards of the dominant group in the gym setting including socially-revered identities and stereotypes, such as competent, fit, strong, healthy and for men, with masculine mannerisms. For example, among 1,500 adults exercising at “health clubs”, overweight participants were more embarrassed, more intimidated when exercising, and less comfortable around fitter others than non-overweight participants (23). In another survey, a general sample of male gym users rated a list of scenarios on how much anxiety they felt: scenarios in which the user did not meet perceived standards of body performance, competence, or appearance were most highly rated for anxiety (24). The top-rated (“someone hovering over me wanting to use equipment”; 33% of sample rated as anxiety-provoking) implies undesired scrutiny by others of performance, competence, or appearance, with other feared scenarios involving not meeting a desired standard such as “spotter had to rush to assist me with the weight” (32%), “someone commented on my appearance” (25%) and “my form was corrected by a trainer” (24%). Some users also rated six further scenarios as anxiety-provoking, including “if I was unable to push the weight”, “if I was lifting weights lighter than everyone else”, and “if the gym was packed”. Thus, gym anxiety or intimidation has been most assessed in those with overweight, disabilities or who are minoritized in some other way, yet is still observed among general gym users.

### What causes gym intimidation?

While there is a profusion of ideas in internet articles aimed at exercisers, only a handful of academic studies have investigated causes of gym intimidation to date. Coulter (17) inferred upward social comparisons to be the cause of intimidation in gym settings, as the presence of exercisers working at greater intensity nearby was found to lead to reduced duration of machine use in one study; of note though, participants did not know they were being observed and intimidation was assumed rather than measured. In a later study, participants viewing a video of cycle racers passing them in a peloton reported intimidation (17). Levinson and colleagues (15) developed a scale to assess social anxiety in exercise settings, implying social anxiety as a mental health disorder causes intimidation for at least some exercisers, presumably those who also experience social anxiety outside the gym. However, the research summarized above that has explored what gym exercise experiences are most intimidating suggest that more is going on.

#### Gyms as places of unequal power

Social power (henceforth referred to simply as “power”) is the extent that members of a group in society can influence others, or get what they want, even if members of other groups do not wish it. According to Russell (25), “the fundamental concept in social science is Power, in the same sense in which Energy is the fundamental concept in physics. Like energy, power has many forms, such as wealth, armaments, civil authority, influence on opinion”. In the gym setting, it is the latter form of power that appears significant, with those who possess socially preferred physical and psychological characteristics (masculine, young, healthy, expert, slim, muscular, extraverted) having the greatest power in that setting.

Conceptualizations of power used by social scientists have varied, but many social scientists today would see power as not necessarily wielded by a fixed entity (e.g., a ruler) but operating at all levels of society and in all social institutions (e.g., a gym), and by all people to some degree, depending on the relationship and social interactions involved; therefore a person's or group's power depends on the situation and can be dynamic, shifting as the situation or people involved change. Power is not necessarily oppressive, but can be; it can also create dominant ways of thinking (*discourses*), which can shape public attitudes especially through the designation of a certain type of person as expert who promotes certain ways of thinking about something, especially when the seemingly “powerless” accept, endorse and spread these attitudes (e.g., psychiatrists portraying certain problems as a mental “illness”). This contextual, dynamic and distributed way of thinking about power derives from the ideas of Michel Foucault (26).

Social science research has explored and critiqued the physical and social features of gyms as places of unequal power. This differs from the body of literature above about situations in which intimidation is experienced, in that this work examines social power in the gym explicitly. Researchers have used qualitative methods to collect data from participants with lived experience of gym exercise, using research philosophies, methods and analytical strategies sensitive to inequalities. Feminist research is one such approach, along with critical approaches. In a study amongst gym users in Sweden, gyms were found saturated with norms defined by masculinity or by high expertise and embodying a hegemonic masculine ideal of behavior and performance (norms being standards for what is typical, expected or proper in a social situation). Participants who did not align with these norms felt deterred from attending, privileging the right of those who met these norms to occupy the space (27). In another recent qualitative study in Sweden, Pelters (28) found various norms based on interviews with diverse, able-bodied, regular users at gyms; most participants deviated from identified norms in some way at some time, with norm breaches causing painful feelings of non-belonging. Pelters argued that because such norms vary depending on area within the gym and who is using that area, gyms are made up of “fluid performance zones” where norms vary depending on who is there at a given time. Using ethnography and interviews with women under 30, Turnock (29) found several ways in which the male body, male performances, or traditional male styles of interaction (e.g., banter) were the standard at four gyms in south-west England. As a result, gym users perceived pressure to conform, with non-conformers feeling tolerated, but not necessarily accepted.

Critical geography researchers have conceptualized gyms as a set of spaces with boundaries defined by possession of preferred characteristics such as masculinity, health, size, performance, or competence. From this perspective, Coen and colleagues (30) found that gym users in the UK not congruent with a dominant masculinity engaged in cognitive wrangling over their legitimacy in gym areas, due to not meeting social norms of that space; users reported sometimes fearing entering the most masculine gym spaces (e.g., free weights area), as it triggered insecurities about their competence and appearance. Transgression of norms for a given space prompted the expectation that others would evaluate the exerciser based on size, performance, and competence.

Gyms have also been conceptualized not just as gendered, but also “figured”, in that power dynamics operate based on the attributes of one's physique, as well as gender; researchers have found some gym users experience place-based exclusion due to a norm in those spaces that users should be healthy, slim, and/or muscular. Among overweight gym users (male or female) in England in early or middle adulthood (31), participants perceived that being healthy and slim conferred higher status than exercisers with overweight or less healthy bodies. One tool to negotiate these structures of power was wearing clothing from socially preferred brands flattering for one's body size. In Singapore, Shee (32) found that users were affected by the gym (or parts of it) being associated with dominant groups, such as certain ethnicities (Singapore being a multicultural nation) or age groups. Users felt either connected or disconnected depending on whether they perceived the gym as associated with a group that excluded them, leading to power geometries between different types of users. This dominance was reinforced even by the type of music played, which appealed more to some ethnicities or age groups than others. For Shee's participants, these power imbalances led to emotions from past gym experiences (such as shame, inferiority and fear) being “stickier” for some users; for example, insecurity about one's body appearance or performance “stuck” more to men from ethnic minorities.

Fisher and colleagues (33) found that within the same gym, some spaces can be “men's spaces” and others “women's spaces”, with norms for appearance and competence generating unease. For young women, access to a women-only section of a mixed gym gave comfort and increased confidence but reduced their “allowed area” even more. Women negotiated the “men's space” by choosing flattering clothing, and preferred gym areas with non-threatening others and easily understood equipment to maximize the appearance of competence.

Norms in the gym can go beyond gender, body attributes, competence or performance; as well as all these, gym users with a physical disability perceived a conflict with a “no pain, no gain” ethos that did not fit their vision of exercise (34), an ethos also found to be a norm among Pelters’ able-bodied participants (28). The participants with a disability experienced more difficulty fitting in at the gym due to accessibility problems (e.g., space for a wheelchair) and actions by other users perceived as othering and invalidating them as a gym user, such as staring or expressing surprise at their presence. Conversely, the physical characteristics of the space can affect who is privileged; one gym studied in England had been a place with little concession to design or appearance of the space and embodied a masculine culture associated with rurality, emphasizing arduousness of the exercise and a lack of focus on appearance, attracting men whose own form of masculinity was a good fit. After a remodeling of the space to fit a “global gym” aesthetic with new lighting, mirrors, and decoration, more women began to use the gym and some of the rural male users no longer fitted in so well (10).

#### The underlying role of social power in gym anxiety

The above body of literature shows that power differentials are experienced between types of gym users, often associated with not meeting norms in that gym space, and the privilege of certain types of users over others. However, it is still not clear why only some gym users experience intimidation despite being members of non-powerful groups in the gym, or why being on the negative end of a power imbalance leads to intimidation. Explanations like this are typically the job of theory: A theoretical framework is currently lacking for understanding how power differentials in the gym might lead to intimidation, why this occurs more for some users than others, and how to intervene.

What appears key in the body of literature above is the operation of significant power imbalances in the gym setting, which for some users lead to threat to one's sense of adequacy of self. The above research implies that it is not upward social comparison (at least, not alone) that is causing gym anxiety, but the experience of *threat* from being on the wrong end of an imbalance of power, when the exerciser feels “different” to those in power in that space.

##### Power is reinforced by meeting or not meeting norms

Social norms are standards for what is typical—*descriptive norm*, or proper—*injunctive norm*—in a specific situation (35). I argue that power in the gym is conferred on some or all other users in that gym, in the mind of the anxious user, by those “powerful” users aligning with injunctive norms and subtracted from the anxious user by not meeting the injunctive nor descriptive social norms of that gym space. However, as Anderson and Dunning (35) point out, injunctive norms can often be descriptive norms at the same time: for example, a belief that most gym users should be, look and perform a certain way, and believing that is also what most gym users are or do (whether the latter is accurate or not), which is different from a norm that one should not drop litter (injunctive norm) while knowing that most people do drop litter (descriptive norm). Hence the distinction between descriptive and injunctive norms may not be relevant for gym anxiety. This distinction between types of norms was not made in most of the research discussed above, and indeed descriptive norms and injunctive norms may have been the same (e.g., “everyone else there is young and fit except me”). It may be that if a majority of users are different to the injunctive norm (i.e., descriptive norm and injunctive norm are different), this may give the anxious user relatively greater power due to numbers. However, if an individual user meets neither descriptive nor injunctive norm, then their power is reduced, but meeting one of these types of norms would be protective (e.g., if you are like most others in your gym, you meet the descriptive norm and would be unlikely to feel intimidated even if not meeting an injunctive norm of society's ideal standard of appearance or fitness).

These social norms are as perceived by the anxious user (and thus descriptive norms may not be accurate), relating to how a body should look and function in that gym; injunctive norms are informed by societal standards of female or male beauty (e.g., slim and toned) or hegemonic masculinity. While descriptive norms are informed by the anxious user's perception (or assumptions) of how most others in that gym space appear (e.g., sex, mannerisms, youthfulness, muscularity, slimness) or perform exercise (intensity, technique, weight used, etc.), among other factors. As they are a perception or assumption, a user's idea of descriptive norms for a given setting may be inaccurate.

The perceived threat to the self may be even more potent where the power imbalance is combined with the powerful having an advantage of numbers, such as when a gym (or part of it) is crowded. Social comparison and social anxiety may therefore not be causes of intimidation, but instead all three are consequences of an underlying process—the operation of social power and thus in turn to what extent the person perceives a resulting psychological or physical threat. Upward social comparison then merely confirms one's non-alignment with these norms as perceived by the gym user—but this upward social comparison and norm violation does not necessarily lead to intimidation and is merely the first step.

### The way forward: the need for theory

With perceived power differentials and intimidation in the gym appearing to be linked, what is needed now is theory. According to Van Lange (36), psychological theories should address the “how” and “why” of a phenomenon, help to reach an underlying truth, identify coherent structures in what appear to be chaotic phenomena, and should point to potential interventions. Importantly, theory should also explain why one person might experience intimidation in the same gym setting when another person does not. Prior research and scholarship summarized above has not used or developed a coherent psychological theory and instead has focused on single constructs such as upward social comparisons [e.g., (17)], power, or perceived social norms of gym spaces. This represents a missed opportunity for a higher-level understanding of the phenomenon of gym anxiety that explains how component processes may be interconnected, processes which may underlie others, and points the way for interventions.

## Introducing the power-threat-meaning framework

I propose that Johnstone and Boyle's (37) Power-Threat-Meaning Framework (PTMF) is the most useful theoretical framework for explaining gym anxiety and intimidation. Clinical psychologists in the UK developed the PTMF to understand mental health experiences in the context of the individual's life within society and individual meaning-making, taking into account that such individuals often have experienced adversities. The PTMF was set out as a potential replacement for a medicalized, diagnostic conceptualization of mental health problems. While originally applied to clinical settings for mental health problems, Johnstone and Boyle argued that PTMF can be applied to any form of emotional distress or problematic behavior, (excluding when there is a clear biological cause such as brain injury, or effects of a drug). This is appropriate to gym anxiety because it is a psychological problem but not typically seen as a mental health problem.

Firstly, I will mention some uses of and research using the PTMF, then present its vision of the person, followed by a description of its framework. I will then discuss the role of its four concepts of power, threat, meaning and threat response, and then use it to outline an explanation of gym anxiety. It should be noted that PTMF could also be used to explain social anxiety or social physique anxiety by the same causes, so any distinction between these and gym anxiety/intimidation may be moot.

### Uses of the PTMF

The PTMF has generated interest among mental health practitioners, with case examples of good practice given at the British Psychological Society's website (https://www.bps.org.uk/power-threat-meaning-framework/good-practice). In research it has been used less often, typically in mental health, forensic psychology, or educational psychology (38). Researchers have used PTMF to explain difficult experiences in groups such as violent prisoners and prison officers (39), adult children of narcissists (40), stress in elite footballers (41), and mental health caregivers (42). Gallagher and colleagues (38) included 17 studies using PTMF in their scoping review, finding that most were qualitative; some studies coded data for presence of PTMF concepts, others structured the collection of the data around PTMF concepts, or evaluated psychological services designed using the PTMF.

### How the PTMF sees the person and psychological problems

The PTMF assumes the person actively processes and makes sense of what happens to them, influenced by meanings and ideas from their society and culture. The image of the person in the PTMF is that all human experience is socially and culturally embedded. Importantly, the PTMF is not intended to reinforce victimhood, passivity or blaming others, but focuses on making explicit the processes which are usually implicit, enabling the individual to take control and make a deliberate choice about how they respond to a given situation.

In the PTMF, psychological problems are seen as understandable (albeit unhelpful) coping or survival reactions to challenging situations, rather than individual pathology. The PTMF conceptualizes psychological problems or symptoms (which in the gym setting could include anxiety, hypervigilance, shame, body self-scrutiny) as “meaning-based threat responses to the negative operation of power” (37)—by *threat response* this means they are seen as survival strategies which are used to cope with or reduce the harm from a potential threat, albeit not always desirable or helpful strategies; for example, anxiety and hypervigilance could keep a person alert to a threat, while shame focuses the individual on not meeting perceived societal standards and to conform so as to avoid ostracism.

#### Unequal power is the root of psychological problems in the PTMF

The social environment often features inequalities of power, which limits and restricts some (e.g., the sufferer) while privileging others who hold power. The PTMF sees life circumstances as “patterned by the operation of power” (43), and that power is the lynchpin between the social environment and personal/psychological difficulties. According to Boyle (43), the key influence on the way that power in the PTMF is conceptualized is the ideas of Foucault as summarized above. The core idea in PTMF is that social *power* is at play in social structures, organizations, the physical environment, media, education, or social relations. Power operates through one or more of several modes, according to the PTMF (43); most relevant to fitness settings are *embodied power* (possession of socially-valued physical attributes, such as muscle mass, low body fat, strength, health), *interpersonal power* (provision, withdrawal or rejection of acceptance or love, friendship), and *ideological power* (control of language, meaning and perspective—such as imposing a neoliberal ideology featuring social comparison, competition, and individual effort). This power operates by creating norms, standards and desires, which then trigger self-surveillance and self-regulation of one's characteristics and actions to fit these norms (43).

In addition, there does not need to be an identifiable traumatic incident that “triggers” psychological problems; according to the PTMF, it is more often the everyday struggle of negative experiences of power, rather than specific, identifiable traumas, which lead to threat and threat responses. In gym settings, such everyday struggle may be about being different to seemingly preferred people, with these everyday struggles “filtered through unquestioned and unrealistic social norms and expectations” (38), which may be about how exercisers in the gym ought to look, act, and perform.

#### Power, threat, and meaning combine to create “symptoms” (threat responses)

The PTMF has four concepts which are interrelated: *power*, *threat*, *meaning,* and *threat response*; as the person is seen in context in the PTMF, the social and physical context can influence any of these four concepts, adding up to what threat responses (if any) are activated. Power is a contextualized experience, and the power of others can be present more or less depending on the setting (e.g., felt in gym vs. absent during home exercise or felt more in one gym than another). The effect of power is that the person self-polices and engages in self-surveillance, adopting the gaze or standards of “powerful” others (43). At times, a mismatch between the person and the expected values and standards may lead to a *threat* as perceived by the person, threats which are often subtle and psychological, but occasionally physical and overt. This threat may take various forms, such as the person's physical or psychological needs no longer being met—such as lack of safety/security or feeling unvalued/ineffective in social roles (43). Threats could also be the possibility of difficult experiences such as stigma, loss, perceived inadequacy, painful emotions, exclusion, shame, humiliation, or invalidation of one's experience or choices. Importantly, the perceived threat may or may not be realistic, and it is the perception that matters. Power can also operate in positive ways, such as when coercive power is used to protect a person from harm, or when interpersonal power is used within a close relationship to provide care and love.

Turning to the third key concept in the theory, the person forms *meanings* about the power situation, influenced by common meanings available within that person's milieu and culture, meanings which may be positive and/or negative. While people form meanings about a range of targets, such as individual significance/mattering, higher values, or connections to the social environment, it is only the latter that are operationalized in the PTMF: additionally, the PTMF focuses on meanings formed in relation to everyday experiences, rather than grand, cosmic experiences (44). These meanings modulate how any threat is perceived and what the response should be; examples of meanings suggested in the PTMF that lead to greater perceived threat and greater psychological problems are “hopeless”, “unworthy”, “isolated”, “unsafe”, “excluded”, “inferior”, and “a failure” (37). The role of personal meaning helps to explain why a given situation produces intimidation in some gym users but not others. Why two people might generate different meanings for the same situation is not clearly theorized in the PTMF and this aspect of the framework is underdeveloped at present. However, the developers of PTMF acknowledged that meanings are not entirely a free choice; they are shaped by discourses, which define what is acceptable or legitimate to say, with discourses being based on Parker's definition of “… a system of related terms and statements that, in constructing an accepted reality, simultaneously reflects and transmits power relations” (44). Meanings are also drawn from the individual's cultural background and influenced by past personal experiences such as adversities, education, as well as concepts and ideas dominant in that culture [i.e., meaning is both made and found according to Shotter (45)]. These meanings can be expressed in narratives (i.e., stories about a person's life or current situation), which are influenced by the narratives they have been exposed to in that person's culture or subculture.

Finally, the psychological problems or symptoms that are the outcome of the above processes are termed *threat responses* — strategies adopted consciously or unconsciously for managing the perceived threat, akin to “symptoms”, chosen unconsciously to suit a given context where the threat is perceived. In gym anxiety these threat responses may be anxiety, avoidance of the gym, hypervigilance, giving up, hyperattention to one's body and how it is perceived by others, thoughts that everyone is looking at you, feeling tense, difficulty concentrating, self-blame, anger, or suspicion about other gym users. These responses are seen as survival strategies in the PTMF, strategies which may or may not be genuinely helpful, but are whatever the person has available for the threat as they understand it (37).

##### Under what conditions does the operation of power lead to threat?

The four concepts in the PTMF are not intended to be a linear sequence or stages but instead are intertwined, with *meaning* acting as the binding or glue that holds the other concepts together. Meanings are generated individually using the past experiences and social and cultural context of the individual; when a power differential is present, negative meanings are more likely to lead to perceived threat. Boyle (43) acknowledges that prior experience is one possible source of meaning, which presumably includes learned experience with that form of power and the group wielding it; for example, a gay man who has had several homophobic experiences around young men displaying hegemonic masculinity may be more likely to form meanings such as “I am unsafe” in a free-weights area of a gym where many of this group are present, than another gay man who has had fewer homophobic experiences.

Secondly, Johnstone and Boyle (37) propose that characteristics of the power differential can increase the likelihood of perceived threat, including exposure being long-lasting or repeated, presence of more than one negative operation of power, when the negative use of power is clearly intentional, or when there are a greater number of potential perpetrators, such as in a busy gym (explaining why many people with gym anxiety find busy gyms the most difficult); thus there is a role for context here.

Thirdly, factors within the person: Johnstone and Boyle suggest that negative operation of power is more threatening at an earlier developmental stage (i.e., youth) or if the person has experienced other adversities (as is common with minorities) and/or has fewer coping responses that they know and can use. The likelihood of threat being perceived is reduced by contextual factors such as having access to resources such as knowledge, social support and social connections with relevant expertise.

Fourthly, Johnstone and Boyle (37) also argue that biologically based threat systems within the person may activate to a greater or lesser extent based on genetic inheritance, epigenetic changes based on life experience, and prior lived experience of chronic adversities, especially in youth. These may create changes in brain structure and function such as greater sensitivity of the HPA axis, greater activation of the fight-or-flight response, as well as systems regulating activation of emotional responses and regulation of arousal. So, the same power differential may lead to a different level of perceived threat depending on the person's life experiences and biology.

## Applying the PTMF to gym intimidation

To summarize the PTMF, the negative operation of social power can be perceived as a threat to the person, in combination with the meaning they make of the situation, leading to threat responses (“symptoms”) as coping or survival strategies. The first aspect is power; a gym user might find themselves violating social norms in that gym space due to competence, body size, physical fitness, gender, age, or masculinity, and therefore different from the type of person they perceive as holding power in that particular gym, or for some other reason, and thus having less social power in that situation. Depending on their relative *power* compared to that of others in that space, they may feel they are at risk of being at the receiving end of the negative operation of power (not necessarily that they are negatively affected, simply at risk). As noted earlier, in PTMF this power can be transmitted via various modalities, including embodied power (those with certain body characteristics being seen as having higher status in a particular place or culture), interpersonal power (to accept and value another person, or reject them), and ideological power (able to impose a vision about what exercise or anything else should be, such as that it should be about radical body transformation, or involve intense effort and a lifestyle that revolves around exercise, or personal growth, or demonstrating one's masculinity, or about having fun or improving mental well-being).

Additionally, the PTMF postulates that the effect of power on a person can be amplified if they experience other negative operations of power in their life, as is the case for many minorities (who may experience inequalities via legal power, economic power, or power by force outside of the gym).

Secondly, as a result of the potential for the negative operation of power, some gym users may perceive a *threat*; that they might be stigmatized (37), feel humiliated, harassed, shamed, excluded, laughed at, feel a failure, or invalidation of their vision of exercise which may be to feel good rather than body transformation. Potential threats may therefore be to identity, body and/or emotions. It is important to note that any such threat may not have happened, or will ever happen, but is perceived as a risk.

Thirdly, perceiving threat may be more likely if the sense the person makes of the situation (*meaning*) is that this gym is not the place for them, or that they are unsafe, unworthy, abnormal, or that only certain people have what it takes to be like the other exercisers they see, or of one's body as inferior or self as failure, or self as lacking discipline. Conversely, certain other meanings may reduce the perceived threat despite the same power disparity—according to the PTMF, the factors influencing individual meanings are idiosyncratic and difficult to predict; the key point is that individuals differ in the meanings made and that the meanings do not arise from free choice, but instead are derived from discourses in the social milieu to which the person has been exposed, and from past experiences.

Fourthly, against this backdrop of perceived threat and negative meaning of the gym situation and one's place in it, *threat responses* (which other perspectives may construe as “symptoms”) may in gym anxiety include any of increased self-scrutiny of one's body, hypervigilance to others in the gym, difficulty concentrating on exercises, anxiety, preoccupation with how other gym users perceive the person, avoidance of the gym or certain parts of the gym, and potentially giving up on one's goals.

It is therefore proposed that the individual combination of being on the weaker end of a power differential, the person's own relative power, and perceptions of threat, in the context of the meaning they make of the situation, explains to what degree one feels a sense of gym anxiety and related “symptoms”. It is to a large degree the implicit communication of how a person should look and act in the gym and the perceived living up (or not) to these standards (often then internalized as self-standards) which gives some users power and takes it from others, which for some users leads to threat, negative meanings, and is therefore central to gym anxiety. This understanding can be used in interventions aimed at alleviating or even reducing the risk of gym anxiety.

### PTMF can be used for one-to-one, or wider group-level interventions

Two levels of intervention are possible using the PTMF for gym anxiety. Firstly, among those who seek help individually from a professional such as a sport and exercise psychologist, the PTMF can be used in assessment and formulation, such as by using the guidance provided in it for interviews to ascertain the role of power, threat, meaning and the person's threat responses as well as their strengths. Alternatively, the PTMF can be used to create large-scale interventions at group level to prevent or reduce gym anxiety, either targeting the gym environment and/or targeting psychological processes via interventions delivered through media or technology (for example, mobile apps) or delivered live in a group. I elaborate on each of these intervention options below.

#### PTMF in individual interventions

Using the PTMF in individual work with a client may lead to a more comprehensive understanding and therefore more effective formulation and potentially intervening by targeting one or more of the four concepts in the framework, such as by re-shaping of the person's narrative of their situation to reach a different meaning and including psychotherapy if relevant. This could be provided one-to-one or in groups and is the same as the primary use of the PTMF in clinical psychology practice.

Practitioners can take advantage of the fact that the heart of the PTMF as used in clinical settings, is developing the client's story; that is, a narrative of what has happened to the person, in context, and the sense they made of it. This narrative is co-developed with the client using interview questions that get to each of the PTMF concepts as well as the client's resources and strengths, which can then be worked into a narrative that explains the presenting problems in context (in this case, gym anxiety). Johnstone and Boyle (37) argue that narratives have a long history of being useful in psychotherapy; by comparing the way a client has storied their life, narratives can highlight that a client has more options for how they see and act in their situation than was previously thought, see their identities more widely, and their values more clearly. It should be noted that for this process to be effective any practitioner will need to form a strong working alliance including agreeing on the shared goals of the work, on the tasks that will be part of their work, and form a trusting bond with the client which includes the practitioner having sufficient interpersonal skills (46), as in any form of psychological practice with clients.

As the practitioner assesses the client and co-constructs the formulation, the overarching PTMF questions need to be further explored and elaborated on during interviews with the client; the core of each question is “What has happened to you?” (power), “How did it affect you?” (threat), “What sense did you make of it?” (meaning), “What did you have to do to survive?” (threat responses), “What are your strengths”, and finally, “What is your story?” (37). The resulting narrative acts as a formulation in itself in that it approximates a tentative, co-developed understanding of the factors causing and maintaining the client's presenting problems.

Like formulation, a PTMF narrative can be used as the basis for choosing relevant psychological interventions. The PTMF's main function is to understand psychological problems and is limited in the solutions and interventions that it recommends. However, Johnstone and Boyle (37) argue that simply creating a narrative may be helpful in understanding one's position, enabling some clients to intuitively move on from the ways they had been acting or feeling previously, as if something has “clicked” and the client is now able to consciously take control in a way they could not before the narrative was generated; for mental health this has been tried in service-user-led groups (47). For some clients, providing a coherent explanation that makes implicit processes explicit may be enough for them to reinterpret the meanings they make of their place in the gym environment, and either be able to exercise despite any intimidation or for it to reduce significantly. For example, Richardson and colleagues (10) interviewed experienced disabled gym users who had exercised as a group in a commercial gym while training to become instructors, and who felt discriminated against at the gym (anxiety or intimidation was not specifically mentioned). Without intervention, this group developed a collective story of themselves not as *being* a problem in the gym but *experiencing* the problem of disablism, of exercising for health rather than to develop high muscle mass, and of continuing to exercise at that gym despite the problems imposed on them by its physical and social environment. Richardson and colleagues argued that this acted as a counterstory to the master narrative of the gym and enabled the group to re-evaluate their social role in the gym and create an affirming identity, supporting their continued participation in gym exercise.

It is important to note that when developing a narrative using the PTMF's suggested questions, distressing experiences from the past may arise (e.g., serious perceived threat during one's youth, which may include abuse). Individual work to develop a PTMF narrative should be conducted either by a professional with appropriate psychological expertise (e.g., licensed psychologist, counsellor or psychotherapist) or if not, there should be a support system available to refer clients to (e.g., licensed psychologist or mental health professional to whom a client can be referred if work on the narrative raises mental health issues that had previously been hidden).

#### PTMF as basis for wider group-level interventions

Interventions on a wider group-level can also be implemented, such as with an exercise group, all the users of a gym, or all customers of a personal trainer or fitness company. Some interventions can even include making changes to the structure and layout of the gym setting, targeting one or more of the PTMF concepts. Table 1 shows suggestions for intervening on each of the PTMF concepts in this manner, some of which need to be implemented by a trained member of a psychological profession (e.g., psychologist, psychotherapist, counsellor, certified mental performance coach, etc.) while others could be implemented by fitness professionals, managers or even exercisers themselves without additional training.

**Table 1:** Examples of interventions at group level, targeting each of the four concepts in the PTMF to reduce gym anxiety.

PTMF concept targeted	Intervention options at group level for each PTMF concept
Reduce the Possibility of negative operation of power	Arrange for those from non-dominant groups to exercise with a friend or small group (increasing their own power by being more numerous) or with an assigned exercise partner/group of similar level (interpersonal power in PTMF)
	Select a gym where power differentials between user and others are minimal.
	Alter gym layout to avoid a space where numerous people of powerful group are clustered together (e.g., distribute free weights equipment around the gym rather than in one area)
	Management, trainers, gym staff, online influencers or other authority figures promote the gym as space for exercise with various goals (e.g., mood boost rather than just fitness transformation; ideological power in PTMF)
	Provide easy-to-access opportunities for users to be taught or learn how to perform exercises competently at no extra charge (e.g., videos on app, wall posters about how to exercise, no-charge sessions with instructors or trainers), increasing own power
	Require all exercisers to wear gym-provided clothing that is modest yet allows freedom of movement (reducing the opportunity to see the physique of other exercisers; embodied power in PTMF)
	Alter gym users’ misperceptions of descriptive social norms in the gym (of appearance, demographics, performance, etc.) as has been done with social norms for how much alcohol or drugs peers consume [e.g., (60, 61); embodied power in PTMF]*
Reduce perceived threat	Desensitization of perceived threat by encouraging formation of accepting relationships between “dominant” group members and non-dominant group members (e.g., buddy scheme, mentorship, introducing new members to experienced members)
	Introduce anti-harassment and member conduct policy with robust enforcement (benign use of legal power in PTMF)
	Re-arrange gym layout to avoid direct sightlines from one piece of equipment to another (benign use of material power in PTMF)
	Reduce visibility by incorporating screened side areas where users can exercise without being seen easily, but still visible by gym staff when passing or via CCTV
	Behavioral experiments to test whether feared threats really occur when entering especially “intimidating” spaces (e.g., free weights area)*
	Recommend distraction by using personal electronic devices (e.g., smartphone, tablet, e-reader) and/or headphones playing sound, to avoid seeing or hearing potential threats [as in (17)]
Promote more positive meanings	Explicit and frequent reminders from an authority related to that gym (e.g., management, personal trainers, corporate advertising) that the gym is a place for all abilities, goals and types of people and a range of reasons to exercise is welcomed (note that simply placing notices on the gym wall may not be sufficient)
	Teach reframing of self as “failure” or “inferior” to self as learner*, with more advanced exercisers as source of modelling/growth mindset* (62)
Replace threat responses with more adaptive responses	Teach established, evidence-supported anxiety-management strategies the user can apply themselves, such as self-talk, imagery, relaxation etc.*
	Teaching skills to maintain concentration (e.g., on an exercise)*
	Teaching skills to focus attention away from self and how self is perceived, toward activities (e.g., lifting a weight)*
	Teach skills for self-managing complex emotions such as shame or anger*, and make individual professional psychological support available for dealing with them (e.g., by referral)

* Should be performed by a licensed professional with appropriate qualifications and expertise, working within an ethical framework, such as a licensed psychologist with appropriate expertise, counselor, psychotherapist, or certified mental performance consultant (CMPC). Interventions without asterisk do not require professional training or licensure.

#### PTMF also explains how existing psychotherapeutic interventions reduce gym anxiety

Successful interventions for gym anxiety are few in the published literature, but practitioners (such as sport and exercise psychologists) currently tend to provide individual psychotherapeutic interventions to clients who request help. Therapeutic approaches used for clients with gym anxiety include cognitive behavior therapy (CBT), acceptance and commitment therapy (ACT), rational emotive behavior therapy (REBT), and compassion-focused therapy (CFT).

While each of these therapies has its own theory and techniques, in terms of gym anxiety they can be seen as working via one or more of the perceived threat(s), meanings made, and/or the threat response(s). For example, CBT techniques that identify and replace negative automatic thoughts about being looked at and judged while exercising could be seen as influencing perceived *threat*. Compassion-focused therapy techniques that increase self-compassion could alter the *meanings* the gym users make about themselves and their presence in the gym, moving away from a sense of shame and inferiority. Relaxation techniques used within various modalities such as grounding and progressive muscular relaxation that target anxiety directly can be seen as managing one or more of the *threat responses*.

Thus, one contribution of using a PTMF approach for theorizing gym anxiety is not only to suggest new interventions, but also to provide an overarching framework that explains why current interventions work at a more abstract level than the theoretical basis of each therapy. By doing so, progress can be made in advancing research on the process of change for psychotherapeutic interventions for gym anxiety, potentially making these existing psychotherapeutic interventions more effective, or better integrated. For example, techniques from various therapies could be chosen and used together to target perceived threat and meanings made, rather than a pure approach based on one type of psychotherapy.

Furthermore, incorporating the PTMF provides additional options for interventions for practitioners because of the PTMF's emphasis on the link between wider social processes and power, and threat response (gym anxiety in this case). Because the PTMF locates the ultimate origin of psychological problems in unfair social situations (the gym in this case), rather than in individual dysfunction and individual solutions which underpin many psychotherapies, interventions could target the gym environment and power structures and perceptions within it. This is not to argue that effective psychotherapeutic interventions for gym anxiety should cease to be used; PTMF adds an overarching layer of explanation and additional options.

### How does gym intimidation decline over time without intervention?

An important goal for a theory of gym anxiety is to explain how some people at first feel anxious in gym settings but less anxious with experience. From a PTMF perspective, change over time is possible without interventions addressing the core concepts of power, perception of threat, and/or changed personal meanings. For example, over time a gym user could acquire greater social power within the gym and be less susceptible to the negative operation of power, such as increased fitness, improved physique, and/or increased competence (embodied power), perhaps now meeting norms in that gym. Alternatively, the user may become less susceptible to the negative operation of power in other ways such as by only attending the gym when there are few other users present and thus being at less of a disadvantage in terms of power, or through increased resources such as access to relevant knowledge about exercising through learning or by working with a personal trainer.

Considering perceived threat, over time a gym user may learn that powerful others are unlikely to pose a threat: this may be via repeated exposure to being in the presence of powerful others without negative consequences (i.e., desensitization), or by forming friendships or becoming acquaintances with them without negative consequences, or it may be through the cognitive realization that threat is unlikely.

Considering personal meanings, with experience a gym user may come across new potential meanings which are more positive (e.g., the idea of growth mindset) or reach new cognitive realizations on their own, leading to a reduced likelihood of threat being perceived in relation to an imbalance of power in the gym. For example, new positive meanings could include that the user is not incompetent or a failure but instead is learning rather than failing, or that it is the environment which is problematic rather than being inferior or not belonging there (as in 10). In sum, as the psychoanalyst Karen Horney (48) observed, “Life itself still remains a very effective therapist” (sometimes), and for some gym users who continue attending despite their anxiety, experience can lead to a shift in the concepts in the PTMF that enable a reduction in anxiety and intimidation.

## Discussion: is all this overcomplicating things?

Adopting the PTMF as a theoretical framework may seem redundant or lack parsimony when a number of obvious psychological processes may be operating. For example, Coulter (17) implicates upward social comparisons as a source of gym anxiety, while a now-deleted blog post by Elias (49) suggested social comparisons, low self-efficacy for the exercises intended, perceived difference to other gym users, and anticipated negative judgements from others, as causes of gym anxiety. To this list may be added the spotlight effect [i.e., actors in a social situation believe their appearance and behavior are noticed more by observers than is really the case; (50)], social physique anxiety, trait neuroticism, and the effect of deviating from social norms. It is plausible that these effects contribute to the strength or manageability of gym anxiety; for example, Coulter (17) found that exercising around others working at higher intensity led to stopping the exercise more quickly. However, the sufficiency of solely listing processes like these as the “cause” of gym anxiety is limited for several reasons.

Firstly, some of these effects are likely to be downstream effects of perceived threat; they only make sense when there is a potential threat of rejection, criticism, exclusion, or humiliation. While the psychological effects above could magnify the perceived severity of any threat, they still occur mostly after a potential threat has been perceived. For example, the spotlight effect is greater when attention is focused on the self because one is more conscious than usual of one's own actions or appearance (51); after a memory task, students in a high social-evaluative condition experienced the spotlight effect to a greater extent than those in a low social-evaluative condition (50), as would be expected during perceived threat. Similarly, the operation of power often implies norms that need to be met to be part of the powerful group, triggering self-scrutiny (43) which means social physique anxiety and upward social comparisons with other gym users would follow (or at least increase) after the perception of threat. Similarly, perceptions of difference to others are not of themselves negative unless being different implies threat of exclusion or the breach of another psychological need.

Secondly, simply listing psychological effects like those above would produce a psychological explanation, but this is typically not sufficient for real-world human problems where a biopsychosocial explanation is needed (52). Biological, psychological and social factors are present in the PTMF and a total separation between biological, societal, and psychological processes is not considered feasible, although biological factors play a smaller role in the PTMF. Nevertheless, PTMF enables a biopsychosocial explanation as it allows a role for biology and social processes, as well as psychological factors. As well as providing a fuller explanation, this is advantageous when considering potential interventions because social processes may be easier to alter (including by altering the gym environment) while effective psychological interventions may be difficult to apply at scale for processes like the spotlight effect or upward social comparisons.

Thirdly, scientific theory is most useful and powerful when it provides a coherent set of ideas, featuring concepts and how they are related. Simply providing a “laundry list” of factors with little specification of how they interact or arise from the situation is generally avoided within psychology and other social sciences. Instead, among other attributes, a good theory links phenomena at a higher level of abstraction, as if from a wider view (36), such as in this case, where cognitive processes such as self-scrutiny are being triggered by perception of threat in the gym setting, which is in turn caused by the negative operation of power, perhaps in cohort with other societal, psychological, and biological processes. Rather than self-scrutiny as an isolated, standalone process, in the PTMF it fits in with other processes and interacts with them. The ability of theory to conceptualize how factors interact with each other is an advantage over “laundry lists” of standalone concepts.

Finally, the psychological processes suggested in the “laundry list” approach above derive from a Western science of psychology which has a much-discussed overreliance on WEIRD participants [Western, educated, industrialized, rich, democratic; (53)], with uncertain relevance to people in general. An advantage of the PTMF is that it provides a framework that is a step back from specific named psychological processes, with such processes able to be “plugged in” to the framework depending on which are relevant in a given society or culture; this avoids imposing Western ideas of threat responses or meanings and allows for those that arise from the person's cultural milieu. This was intentional because the field of mental health in which PTMF was developed also has had long-standing problems with imposing Western-derived concepts (e.g., specific diagnoses) on non-Western cultures which do not always fit well, and a framework with greater global applicability was required. Using PTMF enables explanation of gym anxiety that can be tailored to place and culture, enabling global applicability.

Therefore, rather than offering unnecessary complication, using the PTMF to explain gym anxiety results in more useful theory than a “laundry list” of individual psychological processes, yet enables them to also play a role. PTMF also provides: a structure for thinking about how a range of interventions or changes can operate to reduce gym anxiety; a framework for creating a narrative to re-story gym users’ experiences; and invites us to look more deeply at underlying factors that can foster gym anxiety, and is in accordance with van Lange's (36) TAPAS criteria for theory outlined below.

### PTMF can offer what gym anxiety research and practice needs

Van Lange (36) argued that for a theory to be useful it should: be testable to reach an underlying Truth, provide an Abstraction to explain general principles of the phenomenon, Progress in improving the field's current understanding, and Applicability to create interventions for real-world problems (TAPAS). In this regard, the PTMF is testable in Van Lange's terms, in that it proposes concepts that could either be coded and used to structure qualitative analysis (54) or operationalized and measured in quantitative research and used to predict outcomes. It provides abstraction in Van Lange's framework by proposing general principles that explain individual variability in gym anxiety, and provides progress, moving the field on via an overarching framework. Most importantly, it has applicability and points the way to potential interventions as well as providing explanation for why current psychotherapeutic interventions may or may not be successful. PTMF therefore seems to be a useful theory for this particular applied problem.

### Evaluating the PTMF

While the PTMF is not perfect, and indeed no theoretical framework can be perfect, it fulfils much of what a good theory should do, and its use has the potential to take researchers and practitioners a step further in understanding gym anxiety. Criticisms of the PTMF tend to be around its applicability to mental health problems that have traditionally been given a diagnostic label and whether it is genuinely better than current diagnostic practice; because gym anxiety is not a recognized diagnosis in DSM or ICD, and instead appears to be a problem in living, this critique does not seem to indicate any particular difficulties with using PTMF for gym anxiety.

Conceptual critiques that are relevant outside mental health service delivery include that some of the PTMF's assumptions are difficult to test objectively (e.g., the presence of power differentials), and that its evidence base is limited or has been cherry-picked to suit the ideology of its authors, rather than being based on systematic reviews of literature (55). A further critique is that conceptual refinement of the constructs is needed, especially the meaning construct (56). From a philosophical standpoint, Morgan (57) argued that the concept of meaning in the PTMF downplays important aspects of experience and focuses on behavioral adaptation. This implies that it may be more difficult to use the meaning construct in interventions to reduce gym anxiety, especially as the factors that lead to certain meanings are not clearly specified. Morgan further argued that while the inclusion of power is the PTMF's greatest strength, the PTMF conceptualizes power too narrowly as mainly in relation to threat; this however may not be too much of a limitation for gym anxiety but may indicate a need for further development of the power concept. Another critique refers not to the PTMF itself but to the published research using PTMF: Gallagher et al.'s (54) systematic review suggests that the methods used in qualitative research are reported inadequately and calls for a “deepening of the science” in researching the PTMF's usefulness.

Nevertheless, few theories in the social sciences are uncontested and PTMF offers new possibilities to explain problems in living, such as gym anxiety, without resorting to pathologization of the person.

### Future directions for research

PTMF encourages a wide range of research methods, valuing qualitative and quantitative methods equally (37). As such, testing the usefulness of the PTMF for explaining and intervening with gym anxiety should include qualitative and quantitative research. However, since it emphasizes research philosophies and methods that foreground societal and cultural context, lived experience, and how experiences can interact to magnify their effects, this implies a significant role for qualitative research and non-positivist epistemologies. It is therefore unsurprising that most of the published research so far using PTMF has used qualitative methods, often using the PTMF concepts as a framework for structuring interview questions and/or as a framework for top-down coding and analysis (38). This is because, ideally research using PTMF to understand outcomes should have some capability to include the role of participants’ individual meaning-making, subjective experience and/or personal narratives.

While Johnstone and Boyle (37) are critical of over-relying on what they term “positivist” research, PTMF is compatible with the realist epistemology that is the basis for most quantitative and some qualitative research. From a realist perspective, it would be a helpful next step to collect qualitative data about gym users’ experiences of anxiety and intimidation, and code these for the presence of the four PTMF concepts, followed by bottom-up coding to develop additional themes in the participants’ accounts that are not part of the PTMF. This method was used to identify additional aspects of the experience of violent prisoners and prison guards in Ireland beyond the PTMF concepts (39). Another option is template analysis (53) using the PTMF constructs as part of an *a priori* initial coding frame, as used by Leeming and colleagues to explore the impact of the COVID pandemic on those with long-term mental health problems (55).

A second line of research could examine the implications for the practice of using a PTMF approach for clients who present with gym anxiety. There are examples in the literature of evaluating clinical services that have been designed around the PTMF, such as the introduction of routine use of PTMF formulation in a mental health unit, where decreased use of restraint and seclusion were observed after implementation (58). This could include qualitative research with clients with gym anxiety where intervention has used a PTMF-informed approach, but researchers could also use quantitative designs such as experimental single-case or N-of-1 designs (59); some N-of-1 designs could be used to test whether a PTMF-informed intervention led to improvement in gym anxiety, such as multiple baseline designs where the outcome is monitored daily, change being expected only when the intervention is introduced, and separate cases are held at baseline while another case begins the intervention (with change expected to occur only in the cases undergoing active intervention).

Thirdly, research can examine the effects of PTMF-based interventions for gym anxiety that are applied on a wider group-level, such as by physical restructuring of a gym setting or psychological interventions applied to a wide group (e.g., all users of a particular gym). Research designs here are likely to be quantitative, perhaps using before/after designs within a single gym and leading to randomized controlled trials with groups of gyms in intervention arms or control arms of the trial. A concern within these would be that several interventions (e.g., from the table above) could be tried and it would be useful to ascertain which of these contributes most (or if their contributions are more than their sum total). Quantitative measures of the PTMF concepts would need to be developed (i.e., psychometric scales) to check that if the intervention were effective, it was via the mediating effect of PTMF constructs. Consideration would be needed when operationalizing PTMF concepts, as power itself may be difficult to measure; it may be possible to operationalize power as a self-report scale based on perceived power imbalance between self and relevant others for a particular activity (e.g., in the gym). Perceived threat could be operationalized as an amount (e.g., none to strong threat perceived). Meaning would require more work, but could be assessed on a positive-negative scale, or a checklist of common meanings. In sum, multiple lines of research with diverse methodologies can be applied to examine the PTMF's explanatory power and usefulness for practice.

Finally, considering some of the critiques outlined above about the conceptual basis of PTMF, regarding its usefulness for gym anxiety, further conceptual development could be undertaken regarding the meaning construct (56, 57). For example, factors that contribute to the generation of individual meanings could be elaborated, potentially increasing the routes available for intervention to help people form more positive and helpful meanings about their experience.

## Conclusion: towards a future of public exercise for everyone who wants it

For many people, gym-based exercise is a helpful means to attain their goals, providing appropriate equipment, helpful staff and shelter from the weather—if only there weren't certain other people around. I argue that gym anxiety or intimidation does not represent a disorder or pathology but an understandable response among some users to the gym environment. Gyms have been found to be places where power is unequal, resulting in an environment that some gym users perceive as threatening and uncomfortable. Yet gym anxiety and intimidation have lacked a theoretical explanation so far and there is relatively limited published research on interventions to reduce it. Applying the Power-Threat-Meaning Framework gives a way forward by providing a theoretical framework to make sense of what is happening at a social and individual level which results in some people feeling intimidated in gym settings. Rather than indicating individual psychopathology, a PTMF approach suggests gym anxiety is an automatic response to survive and cope in a difficult and perhaps psychologically unsafe social environment. The PTMF can explain gym anxiety by asserting that the presence of power, in combination with meanings the person forms, social narratives and prior lived experience, may lead to perception of threat to one's psychological needs; threats such as invalidation, rejection, ostracism, or violence. Threat responses are deployed automatically and often unconsciously by the person as a survival strategy, including anxiety, hypervigilance, and avoidance. Based on this understanding, PTMF leads to ideas for simple or complex interventions that can be applied to one or more of these four concepts. The result, if researchers, practitioners, fitness professionals and managers go down this path, could be to make the gym experience maximize equality and autonomy, promote confidence and growth rather than anxiety, and make gyms appealing rather than to be avoided for as many varied people as possible.

## Data Availability

No datasets were generated or used for this article. All original contributions presented in the study are included in the article. Further inquiries can be directed to the corresponding author.
